# Supplementary material for: Increasing efficiency of high numerical aperture metasurfaces using the grating averaging technique
Source: Sci Rep. 2020 Apr 28;10:7124. doi: 10.1038/s41598-020-64198-8 (PMC7188898; doi:10.1038/s41598-020-64198-8)
Supplement: Supplementary file 1 — Supplementary Information. [file 41598_2020_64198_MOESM1_ESM.pdf]

Supplementary information for

Increasing efficiency of high numerical aperture metasurfaces using the grating averaging technique

Amir Arbabi,[1,*] Ehsan Arbabi,[2] Mahdad Mansouree,[1] Seunghoon Han,[3] Seyedeh Mahsa Kamali,[2] Yu Horie,[2] and Andrei Faraon[2,†]

[1]*Department of Electrical and Computer Engineering, University of Massachusetts Amherst, 151 Holdsworth Way, Amherst, MA 01003, USA*

[2]*T. J. Watson Laboratory of Applied Physics, California Institute of Technology, 1200 E. California Blvd., Pasadena, CA 91125, USA*

[3]*Samsung Advanced Institute of Technology, Samsung Electronics, Samsung-ro 130, Suwon-si, Gyeonggi-do 443-803, South Korea*

SUPPLEMENTARY NOTE

Here we show that the deflection coefficient of a beam deflector can be estimated using (1). Consider the aperiodic beam deflector shown in Fig. 1e. Assume that the beam deflector is composed of a large number of extended cells and is illuminated with a normally incident plane wave with a power amplitude of 1. Depending on the polarization of the incident light, the deflected light is either TE or TM polarized. The power amplitude of the deflected light A along the deflection angle θ is related to the Fourier component of the electric or magnetic fields of the transmitted light on the output aperture of the device (dashed red line in Fig. 1e) at the spatial angular frequency of $k_0 \sin(\theta)$, and can be found as [1]

$$A = \frac{C}{L} \int_0^L F(x) e^{jk_0 \sin(\theta) x} \mathrm{d}x, \tag{1}$$

where L is the length of the beam deflector. For TE-polarized incident light $F = E_y$ and $C = \sqrt{\frac{\cos(\theta)}{2Z}}$ is a constant that relates the electric field amplitude to the power amplitude, and Z is the wave impedance in the $z > 0$ region. For TM-polarized light $F = H_y$ and $C = \sqrt{2Z\cos(\theta)}$. The deflection efficiency is the square of the modulus of the power amplitude and is given by $\eta = |A|^2$. Assuming that the beam deflector is composed of M extended cells and each extended cell has a width of Λ, we can rewrite (3) as a sum of integrals over extended cells as

$$A = \frac{C}{L} \sum_{p=0}^{M-1} \int_{p\Lambda}^{(p+1)\Lambda} F(x) e^{jk_0 \sin(\theta) x} \mathrm{d}x. \tag{2}$$

Assuming the beam deflector varies slowly from one extended cell to the next, fields over the pth extended cell can be approximated by the fields of a grating created by periodically repeating the same extended cell. We denote the grating's transmitted field over its output aperture by $F_{\mathrm{g}_p}(x)$. Thus,

$$A \approx \frac{C}{L} \sum_{p=0}^{M-1} \int_{p\Lambda}^{(p+1)\Lambda} F_{\mathrm{g}_p}(x) e^{jk_0 \sin(\theta) x} \mathrm{d}x = \frac{C}{L} \sum_{p=0}^{M-1} I_p, \tag{3}$$

where

$$I_p = \int_{p\Lambda}^{(p+1)\Lambda} F_{\mathrm{g}_p}(x) e^{jk_0 \sin(\theta) x} \mathrm{d}x = \int_{p\Lambda}^{(p+1)\Lambda} F_{\mathrm{g}_p}(x) e^{jk_0 \sin(\theta_{\mathrm{g}}) x} e^{jk_0 (\sin(\theta) - \sin(\theta_{\mathrm{g}})) x} \mathrm{d}x \tag{4}$$

Note that the grating field and $e^{jk_0 \sin(\theta_{\mathrm{g}}) x}$ are periodic with a period of Λ, that is

$$F_{\mathrm{g}_p}(x + \Lambda) e^{jk_0 \sin(\theta_{\mathrm{g}})(x+\Lambda)} = F_{\mathrm{g}_p}(x) e^{jk_0 \sin(\theta_{\mathrm{g}}) x}. \tag{5}$$

Therefore, we can simplify (6) as

$$I_p = e^{jp\Delta\phi_\mathrm{g}} \int_0^\Lambda F_{\mathrm{g}_p}(x) e^{jk_0 \sin(\theta_\mathrm{g})x} e^{j\Delta\phi_\mathrm{g}\frac{x}{\Lambda}} \mathrm{d}x \approx e^{jp\Delta\phi_\mathrm{g}} \int_0^\Lambda F_{\mathrm{g}_p}(x) e^{jk_0 \sin(\theta_\mathrm{g})x} \mathrm{d}x, \tag{6}$$

where we have defined $\Delta\phi_\mathrm{g} = k_0(\sin(\theta) - \sin(\theta_\mathrm{g}))\Lambda$ and used the approximation $e^{j\Delta\phi_\mathrm{g}\frac{x}{\Lambda}} \approx 1$ for $\Delta\phi_\mathrm{g} \ll 1$. $\Delta\phi_\mathrm{g}$ represents the phase shift from one extended cell to the next. I_p can be expressed in terms of the grating diffraction coefficients as

$$I_p \approx e^{jp\Delta\phi_\mathrm{g}} \int_0^\Lambda F_{\mathrm{g}_p}(x) e^{jk_0 \sin(\theta_\mathrm{g})x} \mathrm{d}x = \frac{\Lambda}{C} e^{jp\Delta\phi_\mathrm{g}} t_n(p\Delta\phi_\mathrm{g}), \tag{7}$$

where

$$t_n(p\Delta\phi_\mathrm{g}) = \frac{C}{\Lambda} \int_0^\Lambda F_{\mathrm{g}_p}(x) e^{jk_0 \sin(\theta_\mathrm{g})x} \mathrm{d}x, \tag{8}$$

represents the diffraction coefficient of the nth diffraction order of an nth-order blazed grating that is designed with the phase of $p\Delta\phi_\mathrm{g}$. Plugging I_p from (9) into (5) we obtain

$$A \approx \frac{\Lambda}{D} \sum_{p=0}^{M-1} t_n(p\Delta\phi_\mathrm{g}) e^{jp\Delta\phi_\mathrm{g}} = \frac{1}{M} \sum_{p=0}^{M-1} t_n(p\Delta\phi_\mathrm{g}) e^{jp\Delta\phi_\mathrm{g}}, \tag{9}$$

which is the average of $t_n(\phi_\mathrm{g}) e^{j\phi_\mathrm{g}}$ over different extended cells. Because $\Delta\phi_\mathrm{g} \ll 1$, $M \gg 1$, and $t_n(\phi_\mathrm{g}) e^{j\phi_\mathrm{g}}$ is periodic with a period of 2π, its average can also be computed as an integral over its period as

$$A \approx \frac{1}{2\pi} \int_0^{2\pi} t_n(\phi_\mathrm{g}) e^{j\phi_\mathrm{g}} \mathrm{d}\phi_\mathrm{g}, \tag{10}$$

which is the result presented in (1).

---

* arbabi@umass.edu

† faraon@caltech.edu

[1] R. F. Harrington, *Time-Harmonic Electromagnetic Fields* (Wiley, 2001).
